# Combined PacBio Iso-Seq and Illumina RNA-Seq Analysis of the *Tuta absoluta* (Meyrick) Transcriptome and Cytochrome P450 Genes

**DOI:** 10.3390/insects14040363

**Published:** 2023-04-06

**Authors:** Min Liu, Feng Xiao, Jiayun Zhu, Di Fu, Zonglin Wang, Rong Xiao

**Affiliations:** Guizhou Provincial Key Laboratory for Agricultural Pest Management of the Mountainous Region, Institute of Entomology, Guizhou University, Guiyang 550025, China; liumin8796@163.com (M.L.);

**Keywords:** *Tuta absoluta*, Illumina RNA-seq analysis, PacBio Iso-Seq analysis, cytochrome P450

## Abstract

**Simple Summary:**

*Tuta absoluta* (Meyrick) is an invasive pest that is extremely difficult to control. It exhibits varying levels of resistance to many insecticides. The use of compound insecticides has emerged as a new choice due to their ability to enhance insecticidal activity and delay the increased rate of resistance. The combined sequencing technology of PacBio Iso-Seq and Illumina RNA-seq is very beneficial to perform the in-depth exploration of its life activities. In this study, we obtained full-length transcriptome and comparative transcriptome data for *T. absoluta* by using the combined sequencing technology, and 21 P450 genes related to abamectin and chlorantraniliprole complex metabolism were excavated and the phylogenetic tree was constructed. Eight upregulated P450 genes were verified by qRT-PCR. It is speculated that these genes are closely related to the response of *T. absoluta* to abamectin and chlorantraniliprole complex.

**Abstract:**

*Tuta absoluta* (Meyrick) is a devastating invasive pest worldwide. The abamectin and chlorantraniliprole complex have become an alternative option for chemical control because they can enhance insecticidal activity and delay increased drug resistance. Notably, pests are inevitably resistant to various types of insecticides, and compound insecticides are no exception. To identify potential genes involved in the detoxification of abamectin and chlorantraniliprole complex in *T. absoluta*, PacBio SMRT-seq transcriptome sequencing and Illumina RNA-seq analysis of abamectin and chlorantraniliprole complex-treated *T. absoluta* were performed. We obtained 80,492 non-redundant transcripts, 62,762 (77.97%) transcripts that were successfully annotated, and 15,524 differentially expressed transcripts (DETs). GO annotation results showed that most of these DETs were involved in the biological processes of life-sustaining activities, such as cellular, metabolic, and single-organism processes. The KEGG pathway enrichment results showed that the pathways related to glutathione metabolism, fatty acid and amino acid synthesis, and metabolism were related to the response to abamectin and chlorantraniliprole complex in *T. absoluta*. Among these, 21 P450s were differentially expressed (11 upregulated and 10 downregulated). The qRT-PCR results for the eight upregulated P450 genes after abamectin and chlorantraniliprole complex treatment were consistent with the RNA-Seq data. Our findings provide new full-length transcriptional data and information for further studies on detoxification-related genes in *T. absoluta*.

## 1. Introduction

*Tuta absoluta* (Meyrick), also known as the tomato pinworm in Southern America, belongs to family Gelechiidae of Lepidoptera. It is a globally invasive pest that is extremely destructive to Solanaceae crops and is difficult to control [1]. The species began in Peru in South America, spread throughout South America in the mid-20th century, and expanded its scope of invasion to Southeast Asia [2,3]. By 2021, *T. absoluta* had spread to seven provinces in northwest and southwest China [4,5,6,7]; thus, controlling its number of occurrences is considerably important. Chemical control is currently the most important method for controlling *T. absoluta*. However, owing to the lack of systematic management and monitoring of insecticide sensitivity in tomato growers, *T. absoluta* has developed different degrees of resistance to pyrethroids, abamectin, spinosad, chlorantraniliprole, and other insecticides [8,9,10]. Compound insecticides have become a novel alternative for chemical control because of their advantages in enhancing insecticidal activity and delaying an increase in drug resistance. Notably, pests are inevitably resistant to any type of insecticide, and compound insecticides are no exception.

Nature is rich in insects. In the face of insecticide selection pressure, insects have developed defensive strategies (behavioral changes, target insensitivity, and metabolic detoxification) to enhance the metabolism of toxic chemicals and ensure survival and reproduction [11]. The cytochrome P450 (CYP) family is important in metabolic detoxification. Exogenous substances such as insecticides often affect the activity of P450 enzymes and induce the expression of P450 genes. With the continuous increase and induction of P450 gene expression, insects enhance their metabolism and detoxification and promote insecticide resistance development [12,13]. The role of the P450 detoxification gene has been demonstrated in various pests, such as *Bemisia tabaci*, *Spodoptera frugiperda*, *Culex quinquefasciatus*, and other species [4,11,14].

With the progress in high-throughput sequencing technology, transcriptome research on insects has become indispensable for understanding their life processes. Currently, short-reading sequencing protocols are widely used for transcriptome research [15,16]. However, because of the limitations of short reading lengths, short-reading sequencers are too short of capturing entire transcripts from end to end, and it is difficult to accurately predict gene structure, limiting the in-depth exploration of biological life activities [17,18]. The emergence of full-length transcriptome sequencing based on PacBio SMRT single-molecule real-time sequencing technology has compensated for the short-reading–long sequencing deficiency. Compared to short-reading sequencing, PacBio sequencing does not interrupt RNA splicing and can directly obtain full-length transcripts. To obtain high-quality data with long reading length and accuracy, researchers have used a combination of Illumina and PacBio sequencing technologies to sequence and analyze species and corrected third-generation full-length transcripts with Illumina sequencing data with high accuracy, reducing costs and saving time. This also results in a better assembly effect. Recently, joint sequencing technology has been widely used in many insects, including *Cotesia vestalis* [19], *Rhynchophorus ferrugineus* [20], *Opisina arenosella* [21]; however, its application in *T. absoluta* is still lacking, providing a direction for us to study the cytochrome P450 family of detoxification genes in *T. absoluta*.

The combination of abamectin and chlorantraniliprole can significantly enhance insecticidal activity and delay the increase in drug resistance; however, pests inevitably develop resistance to insecticides with no exception. In this study, we used Illumina RNA-seq combined with PaBio Iso-seq to obtain the full-length and comparative transcriptomes of *T. absoluta* under stress with different concentrations of the abamectin and chlorantraniliprole complex. This study aimed to explore the P450 differential genes that may be involved in the detoxification and metabolism of compound insecticides in *T. absoluta* and to lay a theoretical foundation for exploring their gene functions.

## 2. Materials and Methods

### 2.1. Insect Culture 

The experimental materials required for transcriptome sequencing were obtained from Baiyan Town, Puding County, Anshun City (Guizhou, China). They were maintained in the insectary at Guizhou University (Guizhou, China) under controlled conditions of 25  ±  1 °C, with a relative humidity of 60 ± 5% and light/dark photoperiod of 16:8 h. Larvae were reared on tomato plants; the host plant was planted in the greenhouse at the Institute of Entomology, Guizhou University; and the adults were fed 10% hydromel (*v*/*v*). The collected insects were brought back to the laboratory for continuous rearing for more than 20 generations and were not exposed to insecticides during the rearing period.

### 2.2. Sample Processing

After preliminary toxicity determination experiments, the virulence regression equation of the abamectin and chlorantraniliprole complex (Syngenta Crop Protection, Nantong, China) was obtained, and the concentrations required for sequencing were determined: *LC_10_* 3.47 mgL^−1^, *LC_30_* 11.86 mgL^−1^, and *LC_50_* 27.791 mgL^−1^. The insecticide solutions were diluted in water containing 0.01% Tween-80 (Tianjin Kemiou Chemical Reagent, Tianjin, China), distilled water, or 0.01% Tween-80 without insecticides as a control. The insecticide was applied via the leaf-dipping method, and a piece of tomato leaf was immersed in different concentrations for 15 s. Forty third-instar larvae were added after drying the leaves. After 24 h of treatment, 15 surviving larvae were randomly selected. This process was repeated thrice. The collected samples were frozen in liquid nitrogen and stored at −80 °C.

### 2.3. RNA Isolation and Sequencing

Total RNA was isolated using TRIGene Reagent (Genstar, Beijing, China). A 1% agarose gel was used to detect RNA integrity and contamination. The concentration and quality were measured using a Nanodrop 2000 spectrophotometer (Thermo Fisher Scientific Inc., Waltham, MA, USA).For Illumina RNA-Seq, 12 libraries with four different concentrations (CK, *LC_10_*, *LC_30_*, and *LC_50_*) of RNA samples were prepared and sequenced. All qualified samples were constructed and sequenced using the Illumina NovaSeq 6000 sequencing platform. For PacBio Iso-Seq, equal amounts of total RNA from each treatment (CK, *LC_10_*, *LC_30_*, and *LC_50_*) were pooled. According to the recommendations provided by the manufacturer, a full-length cDNA library was constructed using the Clontech SMARTer PCR cDNA synthesis kit 2.0 (Clontech, CA, USA), and then the full-length cDNA was amplified by PCR. Its damage and ends were repaired and sequenced on the PacBio Sequel II platform after qualification. The above sequencing was performed at Biomarker Technologies (Biomarker Technologies, Beijing, China). The PacBio Iso-Seq and Illumina RNA-seq data generated from *T. absoluta* are available from the NCBI SRA database under project number PRJNA869533. 

### 2.4. Statistical Analyses

#### 2.4.1. Error Correction and Analysis of PacBio Iso-Seq Data

Sequence data were processed using the SMRTlink 6.0 software. Low-quality reads and artificial sequences such as sequencing primers and adapters were removed, and circular consensus sequences (CCS) were extracted according to the principle of full passes ≥ 3 and sequence accuracy greater than 0.9. Full-length non-chimeric reads (FLNC) were clustered at the isoform level, and full-length transcripts were corrected using Proovread software and Illumina RNA-seq data to improve sequence accuracy. Finally, sequences with high similarity were merged using the CD-HIT software to remove redundant sequences in the transcripts. The resulting transcripts were used for subsequent analyses. We used BLAST software to align all sequences in pairs to predict alternative splicing (AS) candidate events. The alignment results needed to meet the requirements of AS events are as follows: first, the sequence length was greater than 1000 bp, and there were two High-scoring Segment Pairs (HSPs), and secondly, the gap was greater than 100 bp and at least 100 bp away from the 3’ or 5’ end, allowing a 5 bp overlap [22]. Finally, the four most widely used analysis methods (Coding Potential Calculator, Coding-Non-Coding Index, Coding Potential Assessment Tool, and Pfam protein structure domain analysis) for coding potential were used to screen transcripts for coding potential, and the predicted lncRNAs were obtained after filtering the transcripts with coding potential [23,24].

#### 2.4.2. RNA-Seq Analysis of *T. absoluta*

By using RSEM software to quantify the expression level of *T. absoluta* transcripts, using FKPM as an indicator to measure the transcript or gene expression level, and using DESeq2 to perform differential analysis of the samples, in this process, the identified DETs needed to satisfy a fold change ≥ 2 and a FDR (False Discovery Rate) < 0.01, the screening criteria. The screened differentially expressed transcripts were subjected to hierarchical clustering analysis, i.e., by clustering transcripts with the same or similar expression patterns, and finally, functional annotation of the differentially expressed transcripts in the database was performed.

#### 2.4.3. Functional Annotation and Enrichment Analysis of DETs

We used DIAMOND software to annotate it functionally. The obtained transcript sequences were compared to the KEGG [25], Swiss-Prot [26], NR [27], GO [28], COG [29], KOG [30], and Pfam 9 databases to obtain transcript annotation information [31]. ClusterProfiler software was used to perform enrichment analysis of the DETs between the sample groups annotated to the GO and KEGG databases.

#### 2.4.4. Analysis of the P450 Gene

By comparing the transcriptome of *T. absoluta*, multiple sets of differentially expressed genes were obtained. According to the ‘CYP450‘ keyword, differentially expressed P450 genes were searched in major functional annotation libraries, and repetitive sequences were manually deleted. After an NCBI comparison, the selected P450 genes were submitted to the International P450 Nomenclature Committee for formal naming. In this process, to avoid the influence of shorter or incomplete sequences on the accuracy and reliability of phylogenetic trees, we selected P450 protein sequences with complete coding regions and lengths greater than 300 amino acids to perform multiple sequence alignments with Lepidoptera species. Mega7 software was used to construct a phylogenetic tree using the neighbor-joining method (repeated 1000 times).

#### 2.4.5. RNAseq Validation by Quantitative PCR

A total of eight differentially expressed P450s from each treatment group were selected for qPCR validation. The relevant primers and internal reference gene (*elongation factor-1 alpha, EF1α*) information are shown in Table S1. RNA was extracted as described previously. The RevertAid™ Master Mix with DNase I (Thermo Fisher Scientific Inc., Waltham, MA, USA) was used to reverse transcribe these into cDNA. Quantitative PCR reactions were performed on a C1000™ Thermal Cycler PCR (Bio-Rad, Hercules, CA, USA) with a reaction volume of 20 μL per sample, including 1 μL cDNA template, 10 μL 2 × RealStar Green Fast Mixture (Genstar, Beijing, China), 1 μL forward and reverse primers, and 8 μL of ddH_2_O. Finally, the expression levels of the target genes were calculated according to the method described by Pfaffl [32]. 

## 3. Results

### 3.1. Summary of RNA-Seq and SMRT Sequencing Results

On the Illumina Novaseq 6000 platform, we sequenced 12 samples (CK, LC10, LC30, and LC50); the clean data of each sample reached 6.01 Gb, and the percentage of Q30 bases was 92.87% and above. In total, 314,016,128 clean data points (93.71 Gb) were obtained (Table 1). We then mixed the total RNA of the 12 samples, used the PacBio Sequel II platform to sequence the full-length transcriptome of *T. absoluta*, and obtained a total of 75.14 Gb of clean data. The sequencing results on the NovaSeq 6000 were calibrated against the LQ isoform obtained from PacBio, and CD-HIT was used to remove redundant sequences in the transcripts. Finally, 80,492 non-redundant transcripts sequences with an average length of 2174 bp were obtained for subsequent analyses. Among them, 62,762 (77.97%) transcripts were successfully annotated. When we used the non-redundant transcripts measured by SMRT sequencing as a reference to align with the RNA-seq data, 90.75%, 89.22%, 89.09%, and 90.44% (average) of the clean data were mapped to full-length transcripts (Table 1).

### 3.2. Structural Analysis

**Figure 1:**
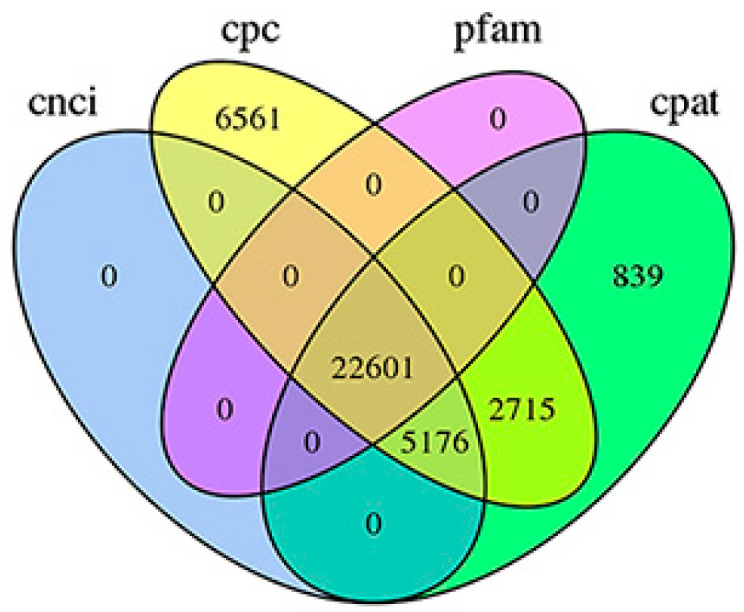
Venn diagram of lncRNA transcripts identified from PLEK, CNCI, CPC, and Pfam. The values in the figure represent the common and non-common parts of each subset.

As *T. absoluta* does not have a reference genome, the type of AS event could not be identified. Therefore, by counting only the number of AS events, 2439 AS were predicted. LncRNAs are a class of RNA molecules that do not encode proteins and are more than 200 nucleotides in length. In total, 22,601 lncRNA transcripts of *T. absoluta* were obtained using four methods: CPC, CNCI, CPAT, and Pfam (Figure 1).

The step-by-step screening method is adopted; that is, the intersection of the prediction results of CPAT and CPC is taken first, then CNCI prediction is performed based on the result of the intersection, and Pfam prediction is performed using the result of the CNCI prediction; thus, most of the Venn diagrams will be 0. The values in the figure represent the common and non-common parts of each subset.

### 3.3. Quantitative Analysis of Transcript Expression

We constructed 12 cDNA libraries from *T. absoluta* and treated them with different concentrations (CK, *LC_10_*, *LC_30_*, and *LC_50_*). The expression level of each transcript was obtained using RSEM, and the overall transcript expression levels and dispersion of each sample are shown in a boxplot (Figure S1). According to Pearson’s correlation coefficient (Figure S2), the correlation between replicate samples was high and could be used for subsequent analyses.

### 3.4. DETs Analysis

After comparing the transcriptome data of *T. absoluta* at different concentrations, the differential transcripts expression results are shown in Table 2. A total of 15,524 DETs were obtained. The results showed that among the six different differential transcripts sets, CK vs. LC10 obtained the most differential transcripts, with 9414, and the number of upregulated transcripts and downregulated transcripts were the highest in all the different sets, with 4146 and 5268, respectively. Compared with the LC30 and LC50 treatment groups, the number of differentially expressed transcripts in the control group was 6892 (3027 upregulated and 3865 downregulated) and 8162 (3581 upregulated and 4581 downregulated), respectively, indicating that many genes in *T. absoluta* respond quickly to insecticide stress. In the LC10 vs. LC30 and LC10 vs. LC50 comparisons, there were 2583 (1609 upregulated and 974 downregulated) and 1848 (1054 upregulated and 794 downregulated) transcripts, respectively. Only 669 (356 upregulated and 313 downregulated) differentially expressed transcripts were between the LC30 and LC50 groups.

### 3.5. Functional Annotation and Enrichment Analysis of DETs

**Figure 2:**
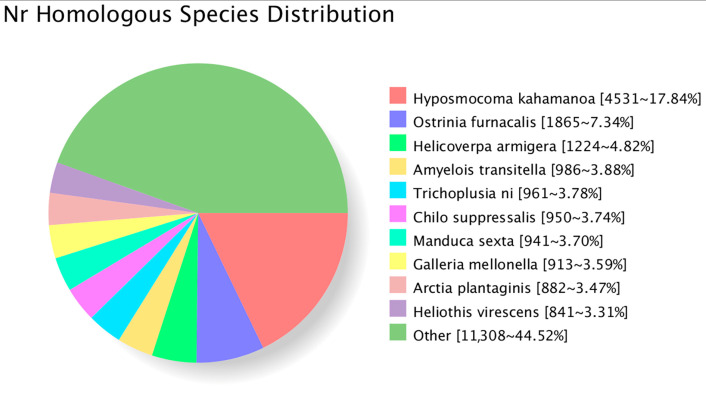
Homologous species distribution of *T. absoluta* annotated in NR.

We annotated the functions of 14,341 DETs in the database. The number of DETs annotated in the major databases is shown in Table 3. NR is a comprehensive database used to identify homologous species using sequence alignments. The annotated DETs in the NR database had the most homologous transcripts with *Hyposmocoma kahamanoa* consisting of 4531 (17.84%), followed by *Ostrinia furmacalis* with 1865 (7.34%) homologous transcripts and *Helicoverpa armigera* with 1224 (4.82%), whereas the other species accounted for 44.52% (Figure 2). 

The GO database is a standard structured biological annotation system. These DETs were classified into three categories using the GO database: biological processes, molecular functions, and cellular components (Figure S3). The GO enrichment results of DETs treated with three different concentrations showed high consistency and annotated 21 subcategories involved in biological processes, mainly cellular, metabolic, and single-organism processes, including protein folding, DNA replication, cell redox homeostasis, and mRNA transport. Annotated to 16 subcategories involved in the cellular component, the annotated transcripts were concentrated in the cell, cell parts, and organelles, including the extracellular region, extracellular fluid, endoplasmic reticulum membrane, and nuclear pores. In contrast, only 11 subcategories involving molecular functions were annotated, and most of the differentially expressed genes were mainly concentrated on catalytic activity, binding function, and transport activity; specifically, oxidoreductase activity, unfolded protein binding, fatty acid synthase activity, and glutathione transferase activity. 

**Figure 3:**
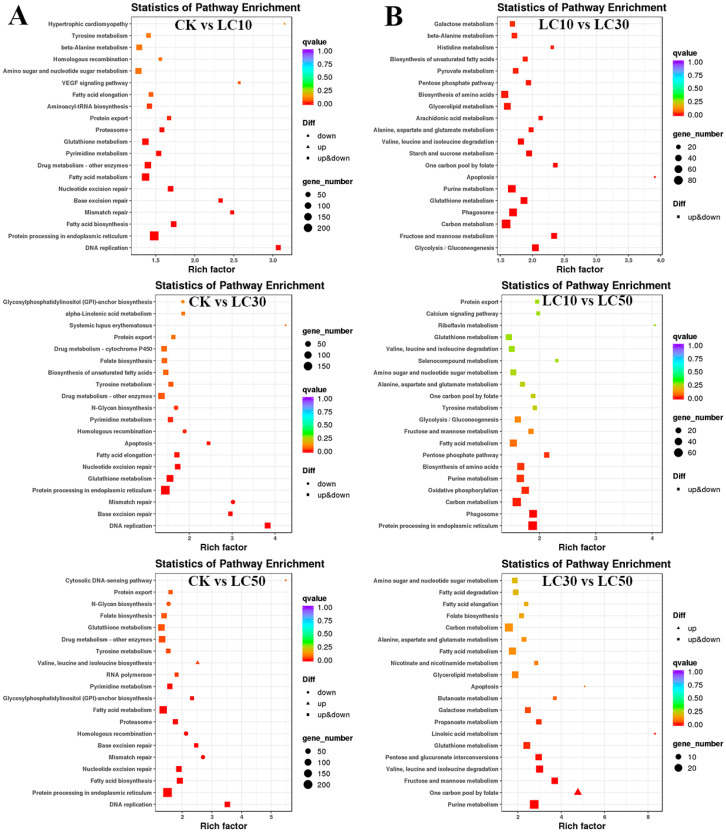
KEGG enrichment analysis of DETs compared between different concentrations. Subfigure (**A**) represents the enrichment results between CK and each treatment group, and subfigure (**B**) represents the enrichment results between the treatment group and the treatment group. The round dot indicates down-regulated genes, the triangle indicates up-regulated genes, and the square indicates both up-regulated and down-regulated genes. The abscissa represents the enrichment factor; the larger the enrichment factor, the more significant the enrichment level of the differentially expressed transcripts in this pathway. The q-value is the *p* value after correction for multiple hypothesis testing. The smaller the q-value, the more reliable the enrichment significance of the differentially expressed transcripts in this pathway.

The KEGG pathway enrichment results showed that in these DET sets (CK vs. LC10, CK vs. LC30, CK vs. LC50, LC10 vs. LC30, LC10 vs. LC50, and LC30 vs. LC50), 208, 197, 201, 153, 144, and 126 pathways were enriched, respectively. In the CK vs. LC10, LC30, and LC50 groups, among the top 20 enriched pathways (Figure 3A), there were 11 common pathways among the three, including protein processing in the endoplasmic reticulum, glutathione metabolism, drug metabolism-other enzymes, and tyrosine metabolism. Using CK vs. LC10 as an example, under *LC_10_* stress, 168 DETs were involved in the fatty acid metabolism pathway. We also identified 112 DETs in the drug metabolism-other enzyme pathway, and 33 DETs were annotated as glucuronosyltransferases and involved in carbohydrate transport and metabolism. Seventeen DETs were annotated as carboxylesterase 2. Eleven DETs were annotated as glutathione S-transferases.

Analysis of the first 20 pathways enriched in the DET sets between the different treatment groups revealed three common pathways (Figure 3B), mainly enriched in fatty acid metabolism, glutathione metabolism, and valine, leucine, and isoleucine degradation pathways. In addition, the LC10 vs. LC30 fractions were enriched in unique fatty acid biosynthesis pathways. The DETs in the LC10 vs. LC50 comparison were enriched in specific pathways such as phagosomes, oxidative phosphorylation, amino acid biosynthesis, glycolysis, gluconeogenesis, and the pentose phosphate pathway. DETs in the LC30 vs. LC50 comparison were enriched in glyceride metabolism, pentose, glucuronic acid conversion, galactose metabolism, propionic acid metabolism, nicotinic acid and nicotinamide metabolism, linoleic acid metabolism, and other pathways. In summary, the pathways related to glutathione metabolism; fatty acid synthesis, degradation, metabolism; and synthesis and metabolism of various amino acids were closely related to the response of *T. absoluta* to different concentrations of abamectin and chlorantraniliprole complex stress.

### 3.6. CYP Analysis

**Figure 4:**
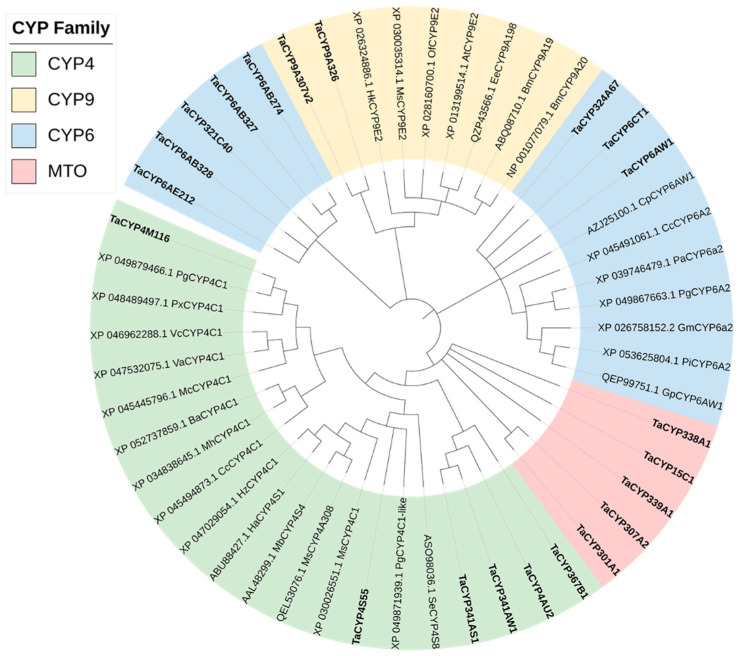
Phylogenetic analysis of 21 P450 genes of *T. absoluta* and related species’ P450s.

In total, 56 differentially expressed P450-related transcripts were obtained from multiple sets of differentially expressed transcripts. Sequences < 300 bp in length that could not be correctly translated were manually removed. After realignment with the NCBI for Biotechnology Information database, 21 differentially expressed cytochrome P450 genes were screened. Among them, 11 P450 genes were significantly upregulated, and 10 P450 genes were significantly downregulated (Table 4). The phylogenetic tree results showed that the 21 P450 genes of *T. absoluta* were mainly concentrated in the CYP4, CYP3 (CYP6 and CYP9 were both branches of the CYP3 gene family), and mitochondrial (MTO) families, which indicates they are evolutionarily conserved and closely related to the lepidopteran insects *Pectinophora gossypiella*, *Mythimna separata*, and *Cydia pomonella* (Figure 4).

Amino acid sequence source: Pg, Pectinophora gossypiella, Vc, Vanessa cardui, Px, Plutella xylostella, Ee, Ephestia elutella, Bm, Bombyx mori, At, Amyelois transitella, Gp, Glyphodes pyloalis, Cc, Colias croceus, Hz, Helicoverpa zea, Ha, Helicoverpa armigera, Va, Vanessa atalanta, Mc, Melitaea cinxia, Ba, Bicyclus anynana, Mh, Maniola hyperantus, Bm, Bombyx mandarina, Of, Ostrinia furnacalis, Hk, Hyposmocoma kahamanoa, Ms, Manduca sexta, Pi, Plodia interpunctella, Gm, Galleria mellonella, Pa, Pararge aegeria, Cp, Cydia pomonella, Mb, Mamestra brassicae, Ms, Manduca sexta, Ms, Mythimna separata, Se, Spodoptera exigua.

### 3.7. RNAseq Validation by Quantitative PCR

**Figure 5:**
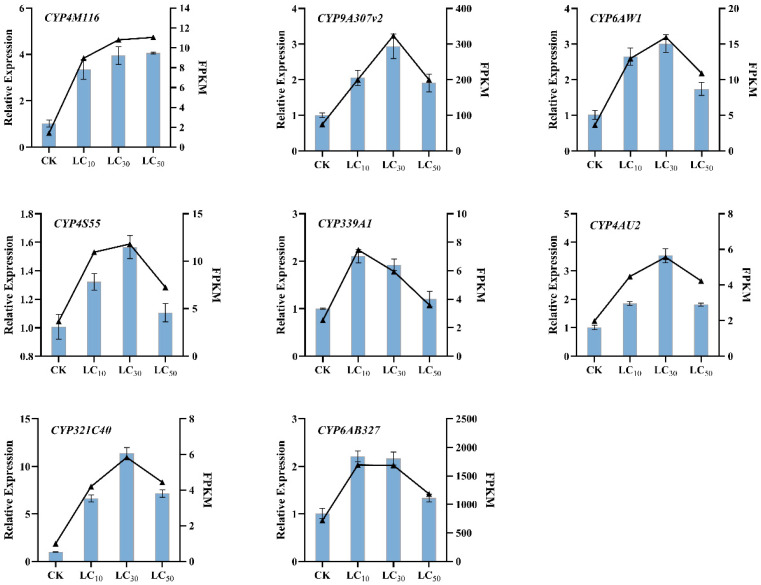
Validation of P450 genes expressions by RT-qPCR.

We chose eight differentially expressed P450 genes to validate the RNA-seq data (FDR < 0.01 and FC ≥ 2) and used RT-qPCR to verify their relative expression levels and trends. The verification results (Figure 5) showed that the relative expression changes of the eight selected P450 genes were highly similar to the trends of transcriptome changes, indicating that the obtained *T. absoluta* transcriptome data were reliable. In addition, except for *CYP339A1* and *CYP6AB327*, the other six genes showed higher expression levels at the sublethal concentration of *LC_30_* 11.86 mgL^−1^, whether by RNA-Seq or RT-qPCR.

Relative expression of the eight genes based on RT-qPCR is represented by a histogram with standard error, and RNA-seq data are represented by a line chart. Each value represents the mean ± SE of three replicates (n = 3).

## 4. Discussion

With the rapid development of sequencing technology, third-generation sequencing technology represented by Pac Bio Iso-Seq combined with next-generation short read length has received extensive attention. Yang et al. [20] used a combination of two sequencing technologies to obtain relatively complete transcriptome data and information when studying *Rhynchophorus ferrugineus*. Joint sequencing technology has gradually been applied to an increasing number of insects. In this study, we combined PacBio Iso-Seq and Illumina RNA-seq to obtain a large amount of transcriptome data of *T. absoluta*, including 80,492 non-redundant transcript sequences with an average length of 2174 bp, much higher than that of *Hypothenemus hampei* (average length of 1610 bp) in Lepidoptera [33], *Carposina sasakii* (average length of 320 bp) [34], and *Mythimna separata* (average length of 416 bp) [35]. 

AS is a central element in gene expression that mediates different biological processes throughout the life cycle of an organism [36] and affects various aspects of protein function, such as binding, localization between proteins and ligands, nucleic acids, or membranes and enzyme properties, which are closely related to the diversity of the gene functions of transcripts [37,38]. PacBio Iso-Seq has an advantage over next-generation sequencing in identifying AS events [39]; therefore, we identified 2439 AS events using PacBio Iso-Seq. LncRNAs are widespread in all eukaryotes and are involved in the regulation of multiple biological processes, including cell cycle progression, cellular differentiation, development, disease mechanisms, metabolism, and immune responses [40]. With the emergence of lncRNA silencing, lncRNAs have been discovered in an increasing number of insect species. Although most of our knowledge of lncRNAs is from *Drosophila melanogaster* [41,42,43], the functional studies of lncRNAs in many insects have recently made great progress. For example, lncRNAs in *Bactrocera dorsalis* may be involved in the regulation of malathion resistance [44]. Regarding regulating immune responses, lncRNAs are involved in the regulation of 20E-induced autophagy in the *Bombyx mori* fat body [45]. The emergence of third-generation sequencing has allowed the identification of more lncRNAs, with 1035 lncRNAs identified in *Bombyx mori* [45], 8096 in *Plutella xylostella* [46], and 3124 in *Zeugodacus cucurbitae* [47]. In this study, we identified 22,601 lncRNA transcripts.

The DETs analysis revealed the metabolic pathways involved in *T. absoluta* under abamectin and chlorantraniliprole complex stress. Many physiological activities began to change when treated with low concentrations (*LC_10_* 3.47 mgL^−1^). To respond to external stress, they must undergo various metabolic activities to enhance their ability to deal with external toxic substances [48]. For example, *Leptinotarsa decemlineata* increases the level of adipokinetic hormones (AKHs, controlling insect energy metabolism) in response to paraquat stress [49]. In this study, after treatment with three different concentrations of the chemicals, the overall gene expression of *T. absoluta* did not change significantly, which may be related to partial gene downregulation. In organisms, many biological functions require different gene products to coordinate with each other, and pathway annotation analysis of DETs can help to further interpret the functions of genes. There were no significant changes in the enrichment results of the GO and KEGG databases among the three different concentrations of the drug treatments, and there was only a significant difference compared to the control group.

Based on this, we mined the DETs and their main metabolic pathways in response to acitretin stress from the KEGG annotation results of the control and drug-treated groups. Insects have long been exposed to a remarkable range of natural and synthetic xenobiotics, and a series of adaptive mechanisms have evolved to deal with these xenobiotics, such as enhancing the biodegradation of xenobiotics for metabolic detoxification [50,51]. As we annotated the results, fatty acid metabolism, drug metabolism-other enzymes, glutathione metabolism, pyrimidine metabolism, and drug metabolism–cytochrome P450 are the main metabolic pathways of *T. absoluta* under stress from the abamectin and chlorantraniliprole complex. Fatty acids generally function as energy storage and structural components of biomembranes and pheromones and as components of defensive secretions in insects [52]. Exogenous insecticides can affect the fat body or nervous system, interfering with the metabolic activity of fatty acids [53].

In addition, in the GO annotation, a large number of genes were enriched in catalytic activity and binding, suggesting that these genes may be related to detoxification metabolic enzymes, such as annotated carboxylesterase 2, glutathione S-transferase, glucuronosyltransferase, and cytochrome P450, which are in *T. absoluta* and play important roles in the metabolic detoxification of the abamectin and chlorantraniliprole complex. Insects respond to insecticide stress with an accompanying increase in metabolism, which is mainly associated with increased detoxification enzyme activity [54,55]. Annotation and analysis of detoxification genes make these transcriptome data powerful corroborators. Numerous studies have shown that herbivorous insects rely on detoxification enzymes to defend against toxic substances and have evolved a series of genes encoding detoxification enzymes, such as P450s, GSTs, and CarEs, which respond to toxic exogenous substances [56].

As one of the largest superfamilies, P450 genes are ubiquitous in organisms; however, their numbers vary considerably. For example, 43 P450 genes have been identified in the arthropod *Neoseiulus barkeri* [57]; however, in the plant *Echinochloa glabrescens*, 233 P450 genes were differentially expressed [58]. In addition, the number of P450 in some model insects is also different. In the same Lepidoptera *Bombyx mandarina*, 68 P450 genes were identified [59]. In contrast, 143 P450 genes have been annotated in *T. absoluta* [60], which is much higher than the number of transcripts identified in this study. This discrepancy may be because their study used fragmented P450 sequences that were retained, aiming to provide all candidate P450s. Notably, to improve the reliability of the results, this study was based on the results obtained after secondary de-redundancy, retaining the amino acid sequence with a complete open reading frame and removing transcripts smaller than 300 aa. Therefore, the number of P450 genes inferred by different analysis methods was not similar in the same species. Although the number of P450 genes obtained by Stavrakaki et al. was different, the results of classifying P450 genes of *T. absoluta* as Clan 4, Clan 3, mitochondrial Clan, and Clan 2 are still consistent with those in this study. Combined with the DETs, 11 upregulated P450 genes were differentially expressed under abamectin and chlorantraniliprole complex stress and these differentially expressed genes were distributed in each cluster.

The CYP6 family is unique to Insecta, and many studies have shown that its members are involved in the metabolism of exogenous and plant secondary organisms [61]. For example, Li et al. [62] demonstrated that CYP6 family genes are related to the resistance of *Bemisia tabaci* to imidacloprid using RNA interference. The CYP6 family also plays a key role in *Locusta migratoria* susceptibility to carbamates and pyrethroids [63]. In addition to CYP6, CYP4 and CYP9 also play important roles in insecticide detoxification. In *Drosophila melanogaster* and *Anopheles gambiae*, more than half of the genes belong to CYP4 [64,65]. In addition, deltamethrin can significantly induce or inhibit *Helicoverpa armigera* CYP6 and CYP9 [66]. In this study, abamectin and chlorantraniliprole complex induced the expression of many CYP4, CYP6, and CYP9 genes, with the strongest induction at *LC_30_*. We speculate that the induction of P450 genes by abamectin and chlorantraniliprole complex is closely related to the concentration. With the increase of concentration, the induction level of P450 genes is also increasing, and the expression level of most genes reaches the peak at *LC_30_*, indicating that *LC_30_* may be the maximum tolerance concentration of these P450 genes to abamectin and chlorantraniliprole complex. Above this concentration, the detoxification ability of P450 genes will be inhibited. These results suggested that CYP4 and CYP3 play important roles in the detoxification and metabolism of *T. absoluta* when stressed by the abamectin and chlorantraniliprole complex. Our results provided an opportunity to identify more *T. absoluta* P450 genes. Although the annotated transcriptome results were analyzed and mined in this study, some transcripts were still not annotated. The study of P450 genes only stayed on simple speculation, and its specific function remains to be further identified.

## Figures and Tables

**Table 1:** RNA-seq data evaluation statistics table.

Sample ID	Total Number of Pair-End Reads	Base Number	GC (%)	Q20 (%)	Q30 (%)	Total Mapped (%)
CK1	29,371,782	8,766,740,538	48.28	98.32	95.46	90.96
CK2	25,534,773	7,623,374,636	48.70	98.23	95.23	91.40
CK3	20,126,926	6,014,056,388	48.63	98.23	95.16	89.89
LC10-1	21,785,788	6,500,875,664	49.22	97.60	93.69	89.60
LC10-2	24,525,334	7,315,451,512	49.14	97.71	93.96	89.28
LC10-3	26,961,547	8,041,765,962	49.06	97.42	93.39	88.77
LC30-1	26,889,981	8,022,376,368	49.69	97.51	93.57	89.55
LC30-2	26,987,086	8,045,001,412	49.43	97.39	93.34	88.60
LC30-3	24,346,249	7,267,996,486	49.40	97.43	93.41	89.13
LC50-1	26,029,165	7,771,009,310	49.82	97.18	92.87	90.12
LC50-2	31,441,769	9,386,493,344	49.59	97.88	94.15	90.83
LC50-3	30,015,728	8,957,881,670	49.44	98.21	95.23	90.38

Total Number of Pair-End Reads: The total number of pair-end reads in clean data; Base Number: The total number of bases in clean data; GC Content: The GC content in clean data, that is, the percentage of G and C bases in clean data in the total bases; % ≥ Q20, the percentage of bases whose clean data quality value is greater than or equal to 20, % ≥ Q30: the percentage of bases whose clean data quality value is greater than or equal to 30. Total mapped (%), percentage of all reads mapped to transcripts in clean reads.

**Table 2:** Statistics of differentially expressed transcripts at different concentrations.

DET Set	All DET	Upregulated	Downregulated
CK vs. LC10	9414	4146	5268
CK vs. LC30	6892	3027	3865
CK vs. LC50	8162	3581	4581
LC10 vs. LC30	2583	1609	974
LC10 vs. LC50	1848	1054	794
LC30 vs. LC50	669	356	313

**Table 3:** Annotated DETs number statistics table.

DET Set	Total	COG	GO	KEGG	KOG	NR	Pfam	Swiss-Prot	TrEMBL	eggNOG
CK vs. LC10	8711	2968	7361	7081	6283	8589	7236	5196	8576	7306
CK vs. LC30	6417	2197	5403	5259	4728	6339	5396	3933	6330	5378
CK vs. LC50	7544	2492	6392	6118	5520	7439	6328	4510	7426	6328
LC10 vs.LC30	2383	879	2052	1995	1779	2358	2016	1564	2361	2050
LC10 vs.LC50	1693	626	1421	1357	1198	1666	1404	1074	1665	1420
LC30 vs.LC50	618	244	531	524	467	610	511	425	611	521

NR, Non-Redundant Protein Database. GO, Gene Ontology. COG, Clusters of Orthologous Groups of Proteins. KOG, eukaryotic ortholog. Pfam, Protein family. KEGG, Kyoto Encyclopedia of Genes and Genomes. TrEMBL: Translation of the EMBL. eggNOG: evolutionary genealogy of genes: unsupervised orthologous groups. SWISS-PROT is a manually annotated and reviewed protein sequence database.

**Table 4:** Differential expression of the P450 gene.

Gene ID	Gene Name	Log2FC (CKvsLC10)	Regulated
BMK_Unigene_050674	*CYP367B1*	1.420509266	up
BMK_Unigene_050670	*CYP4M116*	2.035326565	up
BMK_Unigene_072885	*CYP341AW1*	1.022167794	up
BMK_Unigene_051429	*CYP6AW1*	1.689245983	up
BMK_Unigene_001243	*CYP6AB328*	−1.795651346	down
BMK_Unigene_048006	*CYP339A1*	1.429081153	up
BMK_Unigene_005085	*CYP9A326*	−1.229421385	down
BMK_Unigene_001201	*CYP9A307v2*	1.269973516	up
BMK_Unigene_048337	*CYP6AE212*	−1.103023119	down
BMK_Unigene_073841	*CYP4S55*	1.425289144	up
BMK_Unigene_052034	*CYP307A2*	−1.497466918	down
BMK_Unigene_052058	*CYP15C1*	1.28923048	up
BMK_Unigene_070417	*CYP338A1*	−3.524270134	down
BMK_Unigene_073442	*CYP321C40*	1.82095051	up
BMK_Unigene_071946	*CYP301A1*	−1.421794225	down
BMK_Unigene_096077	*CYP6AB327*	1.106535669	up
BMK_Unigene_093562	*CYP6AB274*	−4.027739403	down
BMK_Unigene_020691	*CYP4AU2*	1.013224445	up
BMK_Unigene_094349	*CYP6CT1*	−3.306571743	down
BMK_Unigene_096172	*CYP341AS1*	−1.916819856	down
BMK_Unigene_096856	*CYP324A67*	−1.304952081	down

Log2FC, Difference multiple logarithm value; up, upregulated gene; down, downregulated gene.

## Data Availability

The data presented in this study are openly available in NCBI SRA database (https://dataview.ncbi.nlm.nih.gov/object/PRJNA869533?reviewer=ikjih8ij3gupsg5ipnd3pgjtm4, accessed on 1 May 2022) at project number PRJNA869533.

## Supplementary Materials

The following supporting information can be downloaded at: https://www.mdpi.com/article/10.3390/insects14040363/s1, Figure S1: FPKM boxplot of each sample; Figure S2: Heat map of expression correlation between pairwise samples; Figure S3: GO annotation classification chart of DEG se; Table S1: Primer sequences of quantitative real-time PCR.
