# Supplementary material for: Combined PacBio Iso-Seq and Illumina RNA-Seq Analysis of the Tuta absoluta (Meyrick) Transcriptome and Cytochrome P450 Genes
Source: Insects. 2023 Apr 6;14(4):363. doi: 10.3390/insects14040363 (PMC10146655; doi:10.3390/insects14040363)
Supplement: Supplementary file 1 [file insects-14-00363-s001.zip › insects-2314733-supplementary.pdf]

Supplementary information

Additional images

S1

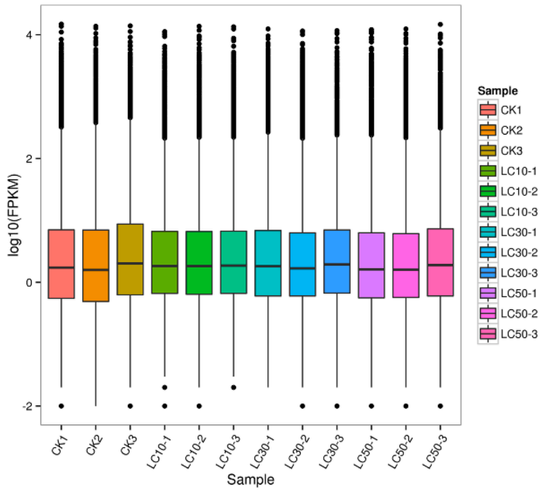

Figure S1 FPKM boxplot of each sample

(The ordinate represents the logarithm of the FPKM expression level. This figure shows the expression level of each sample from the perspective of overall dispersion of the expression level)

S2

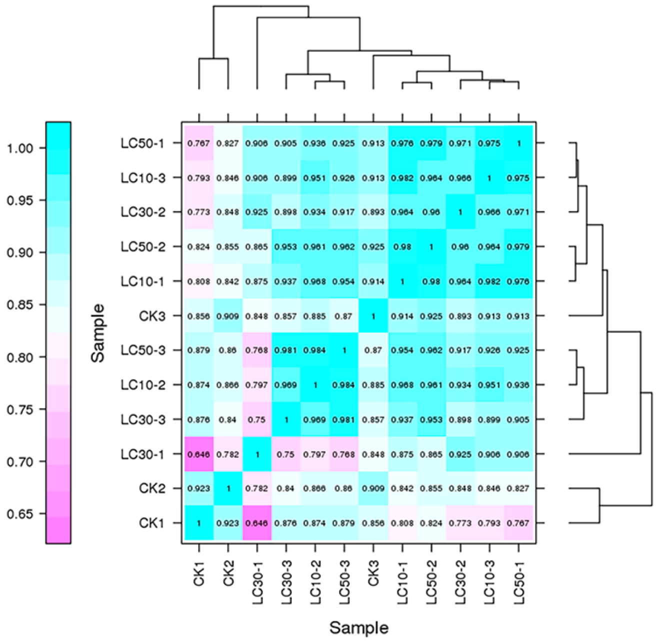

Figure S2 Heat map of expression correlation between pairwise samples

S3

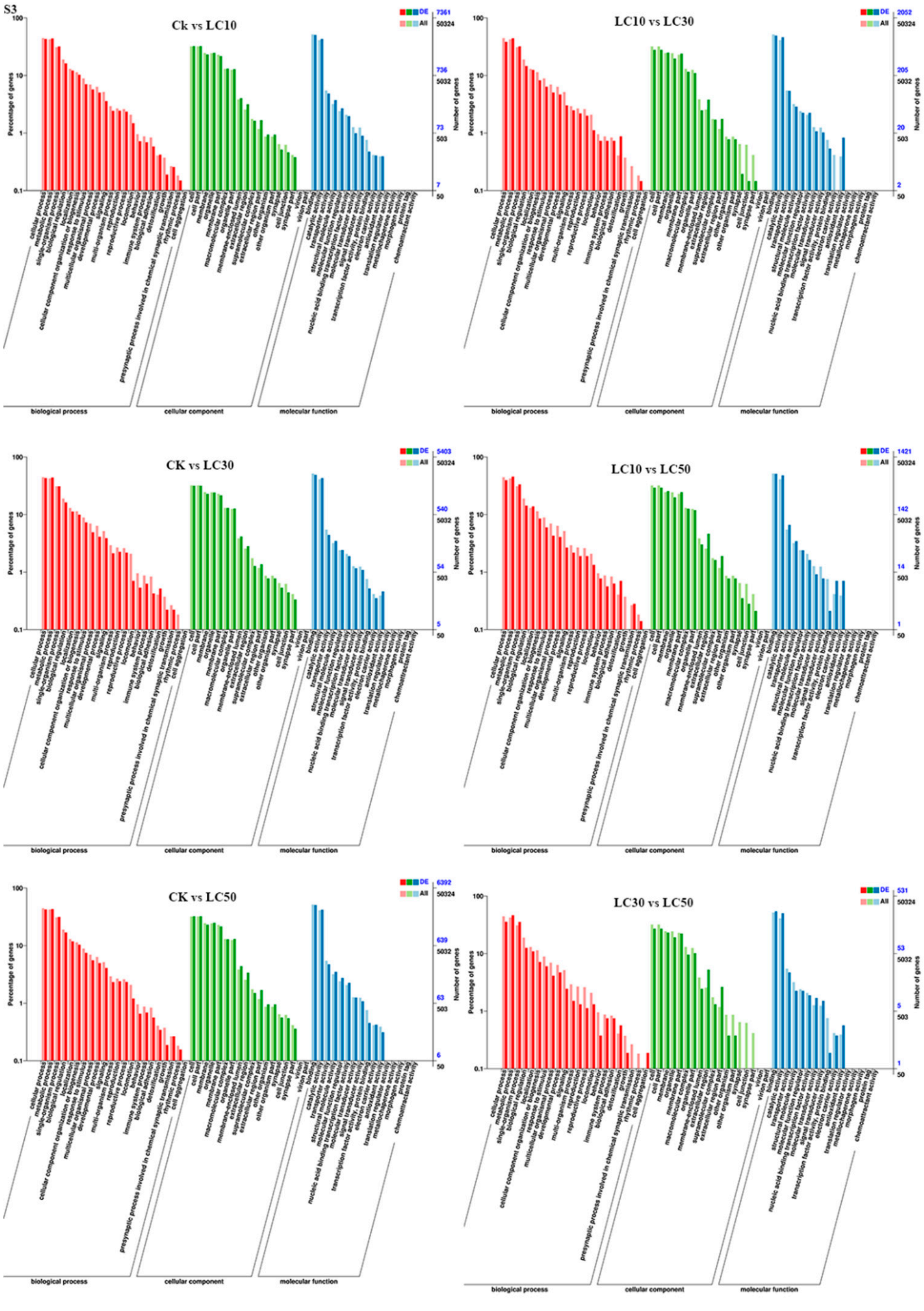

Figure S3 GO annotation classification chart of DEG se

Additional table

Table S1 Primer sequences of quantitative real-time PCR

Gene ID	Gene name	Direction	Primers sequences
BMK_Unigene_050670	*CYP4M116*	F	GACGCCAACTTTCCACTTCAAC
		R	GCCCATCGCTGTTTCGCATA
BMK_Unigene_051429	*CYP6AW1*	F	GCCTTGAAACATCAGCCACAAC
		R	GTCAATCCGTCGTGCTTACTCA
BMK_Unigene_048006	*CYP339A1*	F	TCTCGCTTCACCTCGTCCTG
		R	CGAACGGCAGAACCATAGACTC
BMK_Unigene_001201	*CYP9A307v2*	F	AAAGGTTCGTGGGCAGATTCG
		R	TCGTTCAGGAAGTCTCGGTGAT
BMK_Unigene_073841	*CYP4S55*	F	GGTTCCACGAGAGCATCTATTCA
		R	CGAGAGCACCACCTCAACATC
BMK_Unigene_052058	*CYP15C1*	F	GCAGCAGGAGATAGATGAAGTCA
		R	CACGGAGGATATGCGAAGAGTT
BMK_Unigene_073442	*CYP321C40*	F	GGAATGAGATACGCACGACTACA
		R	CACCGCTTGCTTGCTGTACT
BMK_Unigene_096077	*CYP6AB327*	F	AAGGTGCTCTAGTGGGAGAATCT
		R	AATCCTGCGGCGAAGAATACAA
	EF1α	F	CCCATACAGTGAATCCCGTTTCG
		R	TTGTCTCCGTGCCATCCAGAA
